# Supplementary material for: Associations of Circadian Eating Pattern and Diet Quality with Substantial Postpartum Weight Retention
Source: Nutrients. 2019 Nov 6;11(11):2686. doi: 10.3390/nu11112686 (PMC6893719; doi:10.3390/nu11112686)
Supplement: Supplementary file 1 [file nutrients-11-02686-s001.pdf]

Table 1. Correlations between eating patterns and covariates.

Eating patterns	Age	Ethnic-Malay	Ethnic-Indian	Education-Post-sec	Education-Tertiary	Parity	Night shift	EPDS score	TEI	BMI	Bedtime	GDM	GWG	Mixed Feeding	Formula feeding
Night-eating	−0.022	0.097	0.142	0.029	0.007	0.020	−0.109	−0.022	−0.066	0.080	−0.107	−0.101	0.048	−0.035	−0.065
Night-fasting 9-10h	−0.106	−0.051	−0.184	0.022	−0.022	−0.037	−0.003	0.092	0.071	−0.053	0.215	0.024	0.032	−0.057	−0.079
Night-fasting 11–12h	−0.092	0.019	−0.082	-0.031	−0.078	0.016	−0.038	0.067	0.079	−0.033	0.253	−0.022	−0.068	−0.067	−0.082
Eating episodes 4x	0.001	0.034	−0.044	0.037	−0.127	0.065	0.060	-0.030	−0.289	−0.015	0.116	0.029	−0.014	−0.083	−0.041
Eating episodes 5x	−0.060	−0.012	0.081	0.053	−0.120	0.030	−0.040	0.016	−0.248	0.051	0.063	−0.026	−0.095	−0.046	−0.010
Eating episodes ≥6x	−0.033	0.008	−0.043	0.044	−0.178	0.018	0.022	0.103	−0.366	0.029	0.008	−0.023	−0.052	−0.062	−0.028
Lower diet quality	0.050	−0.063	0.030	-0.023	0.084	0.057	−0.027	0.025	−0.231	−0.024	0.079	0.053	0.037	−0.007	−0.081

Post-sec, post-secondary; EPDS, Edinburgh Postnatal Depression Scale score; TEI, total energy intake; BMI, body mass index at ≤14 weeks of gestation; GDM, gestational diabetes; GWG, gestational weight gain through 15-35 weeks of gestation.

Table 2. Women's baseline characteristics according to their inclusion status in the present analysis from the GUSTO study (*n* = 1026).

Variable	Included (*n* = 687)	Excluded (*n* = 339)	*P* [a]
Maternal age, years	31.3 ± 5.2	29.1 ± 5.2	<0.001
Ethnicity, *n* (%)			0.168
Chinese	392 (57.1)	173 (51.0)	
Malay	172 (25.0)	100 (29.5)	
Indian	123 (17.9)	66 (19.5)	
Education, *n* (%)			0.099
None/ Primary/ Secondary	243 (35.4)	112 (33.0)	
Post-secondary	216 (31.4)	129 (38.1)	
Tertiary	228 (33.2)	98 (28.9)	
Parity, *n* (%)			<0.001
0	246 (35.8)	166 (49.0)	
≥1	441 (64.2)	173 (51.0)	
Employment status, *n* (%)			0.741
Unemployed	227 (33.0)	110 (32.4)	
Employed	460 (67.0)	229 (67.6)	
Night shift, *n* (%)			0.427
No	656 (95.5)	327 (96.5)	
Yes	31 (4.5)	12 (3.5)	
Physical activity, *n* (%)			0.059
<600 MET-min/week	223 (32.5)	125 (36.9)	
600 to <3000 MET-min/week	343 (49.9)	142 (41.9)	
≥3000 MET-min/week	121 (17.6)	72 (21.2)	
BMI at ≤14 weeks' gestation, kg/m^2	23.6 ± 4.5	23.7 ± 5.6	0.727
Total EPDS score	7.4 ± 4.5	7.8 ± 5.6	0.196
Sleep duration, hours	7.2 ± 1.8	7.3 ± 2.0	0.191
Bedtime, 24-h clock	2312 ± 0105	2315 ± 0129	0.688
Total energy intake, kJ/day (1 kcal=4.186 kJ)	7890 ± 2358	7568 ± 3086	0.074

Values are means ± SDs or *n* (%). GUSTO, Growing Up in Singapore Towards healthy Outcomes; BMI, body mass index; EPDS, Edinburgh Postnatal Depression Scale. [a] Based on independent t-test for continuous variables or Fisher's exact test for categorical variables.

Table S3. Maternal eating patterns during pregnancy and substantial postpartum weight retention (≥5 kg) at 18 months based on complete case analysis (*n* = 653).

	Substantial PPWR ≥ 5 kg	
Eating patterns	n	OR (95% CI)[a]
Day-night eating		
Day-eating	568	1.00
Night-eating	85	2.00 (1.04, 3.84)
Night-fasting intervals		
<9 hours	176	1.00
9–10 hours	253	0.81 (0.45, 1.48)
11–12 hours	224	1.17 (0.61, 2.23)
Eating episodes per day		
≤3 times	199	1.00
4 times	209	1.04 (0.55, 1.97)
5 times	136	1.13 (0.53, 2.41)
≥6 times	109	1.71 (0.72, 4.04)
Diet quality		
Higher	327	1.00
Lower	326	1.80 (1.07, 3.02)

Analysis was performed using multivariable binary logistic regression model. All eating patterns were included in a single model and mutually adjusted to each other. PPWR, postpartum weight retention; OR, odds ratio; CI, confidence interval. [a] Adjusted for maternal age, ethnicity, education, parity, night shift, total Edinburgh Postnatal Depression Scale score, total energy intake and body mass index at ≤14 weeks of gestation.

Table 4. Effects of maternal day-night eating and diet quality on substantial postpartum weight retention (≥5 kg) at 18 months based on complete case analysis (*n* = 653).

	Substantial PPWR ≥ 5 kg	
Eating patterns	*n*	OR (95% CI)[a]
Day-eating + Higher diet quality	298	1.00
Day-eating + Lower diet quality	270	1.87 (1.07, 3.25)
Night-eating + Higher diet quality	29	2.37 (0.78, 7.20)
Night-eating + Lower diet quality	56	3.43 (1.45, 8.11)

Analysis was performed using multivariable binary logistic regression in a single model. PPWR, postpartum weight retention; OR, odds ratio; CI, confidence interval. [a] Adjusted for maternal total energy intake, body mass index at ≤14 weeks of gestation, age, ethnicity, education, parity, night shift, total Edinburgh Postnatal Depression Scale score, night-fasting intervals and eating episodes per day.
